# Primary care knowledge and beliefs about physical activity and health: a survey of primary healthcare team members

**DOI:** 10.3399/bjgpopen17X100809

**Published:** 2017-04-19

**Authors:** Patrick C Wheeler, Ralph Mitchell, Melvinder Ghaly, Kim Buxton

**Affiliations:** 1 Head of Service and Consultant in Sport and Exercise Medicine, Department of Sport & Exercise Medicine, University Hospitals of Leicester NHS Trust, Leicester, UK; 2 Senior Lecturer and Visiting Fellow, School of Sport, Exercise & Health Sciences, Loughborough University, Loughborough, UK; 3 Specialty Doctor in Sport and Exercise Medicine, Department of Sport & Exercise Medicine, University Hospitals of Leicester NHS Trust, Leicester, UK; 4 GP, Station View Health Centre, Hinkley, UK; 5 GP, Woodbrook Medical Centre, Loughborough, UK; 6 Assistant Director and Programme Manager, Adults and Healthcare, British Heart Foundation National Centre for Physical Activity and Health, School of Sport, Exercise and Health Sciences, Loughborough, UK

**Keywords:** primary health care, general practice, physical activity, professional practice, health promotion

## Abstract

**Background:**

Physical activity has numerous health benefits and the primary healthcare team are ideally suited to promote activity. The Royal College of General Practitioners (RCGP) has announced physical activity to be a clinical priority in the next few years. However little attention is given to this in medical training, with unclear levels of knowledge and confidence.

**Aim:**

To explore the primary healthcare team knowledge of the benefits of physical activity in preventing and treating ill health.

**Design & setting:**

Questionnaire-based study, from six East Midlands sites in the UK.

**Method:**

Self-completed anonymised questionnaire.

**Results:**

Three hundred and two results were obtained in total, from 166 GPs, 65 GP registrars, and 71 practice nurses. There was a mean age of 44.8 years (range 22–71), with 62% female responders. Fifty-five per cent of responders underestimated UK recommended activity guidance. Responders considered activity promotion as part of their professional role, but this was discussed about one-third as often as other health promotion behaviours, such as weight or smoking. Barriers reported were lack of time (91.2%) and resources (36.8%).

**Conclusion:**

This study has shown reasonable knowledge of recommended levels of activity and accrued health, but most underestimated UK guidance, suggest inadequate levels of activity for optimal health may be being recommended. Confidence in this area is lower in GP registrars than GPs which may mirror other health problems. There was a poor recognition of simple tools to assess the level of physical activity, and low levels of onward signposting or recommendations. If physical activity is to be a clinical priority area of the RCGP, then further opportunities for professional development may be required.

## How this fits in

Physical activity has a wide range of significant health benefits in the prevention and treatment of disease. The RCGP has announced that physical activity and lifestyle will be a clinical priority area for the next 3 years. It is unclear what the level of knowledge and beliefs around physical activity and health exist in UK primary health care, and this study seeks to begin to explore this.

## Introduction

Physical activity is an important and modifiable health behaviour, yet it is one that traditionally receives scant attention in health care. Physical inactivity has been recognised as the fourth leading cause of death globally,^1^ with physical activity having a range of health benefits across multiple disease states. This includes significant benefits in both prevention and management in type 2 diabetes, cardiovascular disease, cerebrovascular disease, hypertension, mental health functioning, and several types of cancer.^2–4^ Despite the recognised benefits to health, the level of medical training dedicated to physical activity remains low. Only 56% of medical schools teach the Chief Medical Officer (CMO) guidance for physical activity,^5^ and final year medical students have been shown to significantly underestimate the health benefits of physical activity,^6^ suggesting that primary care may be underpreparing the doctors of the future, in using this vital tool in disease prevention and treatment.

Studying this area in primary care is not new. A study in Bradford nearly 20 years ago examined GPs knowledge of physical activity and found that they appeared to have high levels of knowledge of the health benefits of regular physical activity.^7^ This study only investigated GPs, and not the views of GP registrars undergoing postgraduate training, or those of practice nurses who have an important role in chronic disease management in UK general practice. A more recent study in Scotland examining the health beliefs of GPs, practice nurses, and health visitors, found that only one-third of GPs correctly identified at least one component of the national guidance relating to physical activity.^8^ Despite this, research has found that medical support provides significant improvement in the adoption and maintenance of an active lifestyle.^9,10^ A recent large multicentre trial promoting activity, specifically to reduce falls through general practice was found to reduce the risk of falling in older adults as well as increase overall level of physical activity.^11^ However, a systematic review in 2011 has shown that while primary care providers were receptive to the concept of counselling patients about physical activity, there were numerous individual and organisational barriers to this being done in practice.^12^

Although some physical activity intervention studies in primary care have shown that there is only a small impact on physical activity, a systematic review and meta-analysis found that promotion of physical activity to inactive adults in primary care significantly increased rates of activity at 1 year.^13^ While physical activity has a large number of health benefits, and activity rates can be influenced by GPs, there remain uncertainties of perceived benefits of activity, and the barriers in practice in the recommendation of physical activity within routine primary care. GPs and other members of the primary healthcare team, have central roles in the promotion of a variety of health behaviours. Their position and potential influence in the local community, and their expertise in the management of chronic diseases, make them ideally positioned to promote physical activities in their communities.

Various strategies have been adopted in the past decade in the UK to highlight the benefits of physical activity for health. These include: the 2009 Let’s Get Moving programme;^14^ the update of the physical activity recommendations 2011 Start Active, Stay Active report from the Chief Medical Officers from England, Scotland, Wales and Northern Ireland^15^ and, more recently, the 2014 Public Health England guidance Everybody Active, Every Day^16^ In addition, in June 2016 Physical Activity and Lifestyle have been announced as clinical priorities for the RCGP over the next 3 years.

Current UK physical activity recommendations state that adults age 19–64 years should aim to be active on a daily basis by performing at least 150 minutes of moderate level activity, such as cycling or brisk walking, or 75 minutes of more vigorous activity per week. They should also undertake activity for muscle strengthening on at least 2 days per week, as well as reducing sedentary time. In addition, adults age ≥65 years, who are at risk of falls, should also include balance training exercises at least twice per week.^15^

It is not clear to what extent the information from the UK Chief Medical Officer report has impacted primary healthcare teams and what level of knowledge, understanding, and physical activity promotion practice exists. This study sets out to identify current knowledge and beliefs about the benefits of physical activity for disease prevention and treatment among GPs and practice nurses in the UK, specifically asking about their own practice, as well as their knowledge of the guidelines.

## Method

Collectively, the authors have given six talks to primary care health professionals about the benefits of physical activity and health. These talks took place between September 2015 to March 2016 and included one Clinical Commissioning Group Protected Learning Time event (CCG PLT), three different GP locality meetings, and two different Vocational Training Scheme (VTS) teaching sessions. Prior to these talks, participants in attendance completed a simple questionnaire which explored their knowledge, understanding of exercise medicine and their practice of promoting exercise to the patients they routinely treat. This questionnaire is based on one developed for another physical activity training project in a deprived London borough and a similar version was used in other research. These questionnaires were completed anonymously and returned before the talks were given.

Data from these questionnaires was collated into an Excel spreadsheet and analysed in SPSS. From this dataset, the majority of the outcome measures are scale or categorical data.

## Results

A total of 316 questionnaires were completed at the six different educational events run by the authors across the East Midlands region in the UK. The meetings were spread across a geographical area, and no responders attended more than one meeting. The type of meetings and number of responders is as follows: CCG PLT (*n* = 196), three different GP locality meetings (*n* = 42, *n* = 11, *n* = 11) and two different VTS (*n* = 23, *n* = 33) Table 1.

**Table 1.** Demographics, health behaviours and knowledge for responders, by professional group

Demographic	All (*n* = 302)	GP (*n *= 166)	GP registrar(*n *= 65)	Nurse (*n *= 71)
**Age, years**				
Mean (SD)	44.8 (10.6)	48.5 (9.0)	32.0 (4.7)	48.1 (8.8)
**Sex, %**				
Male	38	54	35	1.4
Female	62	46	65	99
**Ethnic origin,%**				
White	63	60	37	96
Mixed/multiple ethnic groups	0.3	0.6	–﻿	–﻿
Asian/Asian British	30	36	45	1.4
Black/African/Caribbean/black British	3.7	1.9	9.2	2.8
Other ethnic group	3.0	1.9	9.2	–﻿
**Which of the following best describes your own smoking habits? %**				
I currently smoke 40 or more cigarettes per day	–﻿	–﻿	–﻿	–﻿
I currently smoke between 20 and 40 cigarettes per day	–﻿	–﻿	–﻿	–﻿
I currently smoke under 20 cigarettes per day	1.3	–﻿	1.5	4.2
I used to smoke regularly, but have now given up	11	10	5	21
I have never smoked, or never smoked regularly	87	90	94	73
**Concerning your own weight, do you consider yourself to be? %**				
Very underweight	0.3	–﻿	1.6	–﻿
Slightly underweight	2.7	4.3	1.6	–﻿
About right	49	51	58	37
Slightly overweight	42	40	34	54
Very overweight	6.4	5.5	4.7	9.9
**In the past week, on how many days have you done a total of 30 minutes or more of physical activity, which was enough to raise your breathing rate? %**				
Mean (SD)	2.41 (1.86)	2.36 (1.85)	2.17 (1.72)	2.75 (2.00)
**How many minutes per week of moderate intensity physical activity should an adult undertake to meet the current UK physical activity guidelines? ** (Correct answer is 150 mins moderate activity/week)				
Mean (SD) [range]	122 (81)	128 (96) [0–600]	125 (53) [0–350]	104 (58) [4–250]
**How many days a week should an adult undertake physical activity to improve their muscle strength to meet the current UK physical activity guidelines? ** (Correct answer is 2 days each week)				
Mean (SD) [range]	4.13 (1.31)	4.1 (1.4) [0–7]	4.1 (1.1) [2–7]	4.3 (1.3) [2–7]

Participants were asked to declare their occupation, a total of 166 GPs (partner/salaried or locum), 65 GP registrars and 71 nurses responded. In addition, responses were obtained from three medical students, and one retired GP. Eight responders specified another occupation (including several foundation-level doctors, and a prescribing advisor), and two responders did not give a response for their occupation. Subsequent analysis was performed for the GP, GP registrar, and nurse groups, with the remainder of the responders (*n* = 14) excluded from analysis, giving a sub-group analysis total of 302 responders.

The mean age of these responders was 44.8 years (standard deviation [SD] 10.6, range 22–71); 38% were male, 62% were female, and a single responder provide details of their sex. Using the categories of ethnic origin taken from the 2011 census categories, 63% of overall responders described themselves as ‘white’. To get a better understanding of personal health choices, responders were asked about their own health behaviours, specifically about their smoking status, their perceived weight, and the number of days in the last week that they had accumulated at least 30 minutes of moderate-level physical activity. The majority of responders had never smoked or never smoked regularly, considered themselves to be of about normal weight or slightly overweight, and were active for at least 30 minutes on 2 days of the week. The responses to these questions are displayed in Table 1.

### Knowledge of guidance

Responders were asked about their knowledge of the current UK CMO physical activity guidance regarding the recommended number of minutes per week of at least moderate-level physical activity and the number of days of resistance training for optimal health benefits. Overall, a range of figures were given, with the mean (SD) number of minutes of moderate activity being 122 (81), and the number of days of resistance training specified as 4.13 (1.31). The figures overall, and by professional group, are displayed in Table 1.

In total 55% of all responders were suggesting fewer minutes of moderate-intensity physical activity than the current UK CMO guidance.

### Health promotion behaviours including promoting activity and professional role

Responders were asked if they considered it a part of their professional role to promote physical activity to patients. The vast majority of all the clinical groups reported that they believed that it was, with only two GPs (1.2%) saying that they did not consider it part of their role, and the remainder of the GPs and all of the GP registrars and nurses believing that it was part of their role.

Participants were asked to respond to a series of statements regarding the barriers they perceived were limiting them discussing physical activity with their patients. The responses to this question are displayed in Table 2. The most common reason cited by far was a lack of time responses are presented in order of decreasing frequency.

**Table 2.** Perceived barriers in limiting professionals from discussing physical activity with their patients

	All (*n *= 285), %	GP (*n* = 156), %	GP registrar (*n* = 63), %	Nurse (*n* = 66), %
Lack of time	91.2	91.0	92.1	90.9
Lack of resources	36.8	41.7	38.1	24.2
Patients’ current condition	27.4	26.9	22.2	33.3
Patients are unlikely to follow the advice	24.6	30.1	28.6	7.6
Lack of knowledge	18.9	12.8	39.7	13.6
Lack of incentives	11.2	16.0	7.9	3.0
Not my professional role	2.1	3.8	0	0

To put physical activity promotion into a wider context, responders were asked how often in their routine work they undertook a range of health promotion activities involving potentially modifiable health behaviours or risk factors. There were no significant differences between professional groups seen in the general questions, but the specific questions related to health promotion practice in the physical activity domain revealed that differences were evident between roles. Table 3 displays the responses for the questions across all responders, including the number of responses seen for each professional group.

**Table 3.** Self-reported professional behaviours

	All	GP	GP registrar	Nurse
	(*n* *= *293),%	(*n* *= *159),%	(*n* *= *64),%	(*n* *= *70),%
**How often do you … ‘Ask patients about their smoking habits?’**				
Often	92	96	81	93
Sometimes	7.8	4.4	17	7.1
Rarely	0.3	0.0	1.6	0.0
Never	0	0	0	0
**How often do you … ‘Ask patients about their level of alcohol consumption?’**				
Often	77	77	73	81
Sometimes	22	23	25	17
Rarely	0.7	0.0	1.6	1.4
Never	0	0	0	0
**How often do you … ‘Check a patient's weight?’**				
Often	57	60	25	91
Sometimes	35	36	59	8.7
Rarely	5.8	4.4	16	0.0
Never	0	0	0	0
**How often do you … ‘Check a patient's blood pressure?’**				
Often	94	93	92	97
Sometimes	5.8	6.3	7.9	2.9
Rarely	0.3	0.6	0.0	0.0
Never	0.0	0.0	0.0	0.0
**How often do you … ‘Ask patients about the levels of regular physical activity they are undertaking?’**				
Often	36	34	13	61
Sometimes	54	56	73	33
Rarely	10	10	14	5.7
Never	0	0	0	0
**How often do you … ‘Screen patients using a specific physical activity tool to quantify their activity levels?’**				
Often	11	6.3	1.6	31
Sometimes	29	30	13	41
Rarely	30	35	33	16
Never	30	28	53	11
**How often do you … ‘Signpost inactive patients to local physical activity opportunities?’**				
Often	21	18	6.3	40
Sometimes	54	63	48	39
Rarely	20	16	36	13
Never	5	2.5	9.4	8.6
**How often do you … ‘Provide counselling to motivate inactive patients?’**				
Often	23	24	6.3	34
Sometimes	41	47	33	33
Rarely	25	19	44	20
Never	12	9.0	17	13
**How often do you … ‘Refer inactive patients to exercise programmes?’**				
Often	16	14	6	27
Sometimes	54	60	53	41
Rarely	25	23	31	24
Never	5.5	3.1	9.4	7.1

### Physical activity benefits and measurement

Responders were asked about their use of specific screening questionnaires to measure patients’ physical activity levels. In order to identify any positive-affirmation responses, two fictitious questionnaires were included alongside four genuine and commonly used ones. Results demonstrate a low levels of awareness and use of these physical activity screening questionnaires, with the highest awareness and use being the General Practice Physical Activity Questionnaire (GPPAQ). Fifty-seven per cent of responders stated that they often, or sometimes, used the GPPAQ. This high recognition and use may be a result of it being part of the hypertension Quality and Outcomes Framework (QOF) under the GP contract until April 2014. Despite this, 29% did not know what this questionnaire was. The other questionnaires had much lower levels of recognition, with between two-thirds and three-quarters of responders not knowing about the questionnaires. The figures for different questionnaires, listed alphabetically, are displayed in Table 4 including the two fictitious questionnaires, with no significant differences between the other two genuine and two fictitious questions.

**Table 4.** ‘Which of the following tools do you use to screen patients’ physical activity?’

Questionnaire	Often, %	Sometimes, %	Never, %	I do not know what this is, %
General Practice Physical Activity Questionnaire (GPPAQ)	21	36	14	29
International Physical Activity Questionnaire (IPAQ)	1.4	1.4	18	79
LEAP^a^	1.4	4.7	19	75
PGActiveQ^a^	0.5	2.3	20	78
Single item question	3.2	3.2	17	76
Vital sign	10	6.0	19	65

^a^Fictitious questionnaire, used to identify positive responses.

Responders were asked about their beliefs regarding any benefits that physical activity can have in relation to a range of health outcomes, this included areas where robust evidence exists of benefit and also several questions which were fictitious to identify any positive-affirmation effects. Results are displayed in Table 5, and for clarity are displayed in the order of the proportion agreeing the most with the statement.

**Table 5.** Displaying the proportion agreeing/disagreeing with statements about physical activity and health outcomes

Statement	Strongly agree, %	Agree, %	Neither agree nor disagree, %	Disagree, %	Strongly disagree, %
Physical activity can reduce the risk of cardiovascular disease	65	33	2	0	0
Physical activity can be an effective treatment for depression	62	37	1	0	0
Physical activity can help treat type 2 diabetes	61	37	1	0	0
Adults should minimise the amount of time being sedentary for extended periods of time	57	39	2	2	0
Physical activity can improve mobility and balance	55	43	1	1	0
Physical activity can both prevent and treat lower back pain	38	57	3	0	1
Adults who are physically active have a lower risk of developing certain types of cancer than inactive adults	37	50	11	0	2
Physical activity can significantly reduce hospital admissions for people with chronic obstructive pulmonary disease	28	58	11	1	3
Physical activity can prevent the development of osteoarthritis	25	51	16	1	8
Physical activity can reduce the risk of the development of glaucoma^a^	4	9	60	6	22
The only health benefit physical activity has is in in assisting weight loss^a^	4	2	3	55	37
Physical activity can treat haemophilia^a^	2	2	46	16	33

^a^Thought to be a false answer question, used to identify positive responses

Responders were also asked to self-rate their own level of confidence and knowledge in advising patients about physical activity, both using a 4-part Likert scale. Overall, differences were noticeable between GPs and GP registrars, but this may represent experience and may translate to other disease and therapy areas, or may represent GP registrars being more open about their gaps in knowledge and confidence. Results for these two questions are displayed in Table 6.

**Table 6.** Self-rated confidence and knowledge in advising patients about physical activity by professional group

How would you rate your confidence in giving general advice to patients about physical activity	All (*n *= 302), %	GP(*n* = 166), %	GP registrar(*n* = 65), %	Nurse(*n* = 71), %
Extremely confident	9.7	14	1.6	7.2
Very confident	50	48	33	70
Not very confident	40	37	65	22
Not at all confident	0.7	0.6	0	1.4

Do you feel that you have sufficient knowledge to advise patients about physical activity	All (*n *= 302)	GP (*n *= 166)	GP registrar(*n *= 65)	Nurse (*n *= 71)
Yes, lots	10	15	0	10
Yes, some	62	62	59	65
No, not really	27	22	41	23
No, not at all	1.0	1.3	0	1.4

## Discussion

### Summary

Overall, this study has found that the primary healthcare teams’ knowledge about the recommended levels of activity and the health benefits that these can accrue, was at the right level. The self-reported knowledge of minutes of physical activity was close to UK CMO guidelines, but a higher number of days of resistance/strength training was suggested than current guidance. Fifty-five per cent of responders reported a figure of activity lower than the current UK CMO guidance, suggesting indequate levels of activity for optimal health may be being recommended.

If the RCGP plan for physical activity to become a clinical priority is to move forward, then GPs and practice nurses will need to feel confident in raising the issue with patients, and have knowledge about what to do with the results. Encouragingly, the vast majority of the responders believed that it was a part of their professional role to discuss physical activity with their patients. However, this study has shown that the subject of physical activity is far less likely to be raised than for other modifiable health issues, such as smoking or blood pressure. In this study, only one-third of professionals often asked about physical activity, compared to 90% for smoking. The reasons for this remain unclear, although a number of barriers were identified by this group. Lack of knowledge and lack of confidence may remain an issue, especially with GP registrars, and focused efforts to address this are needed. Furthermore, it was found that rates of counselling for physical activity motivation, referral to exercise programmes, or signposting to resources remain relatively low, suggesting that professionals may not be aware of, or make the most use of, local resources to support patients in this behaviour change. Further education across all professional groups may be needed.

Regarding perceived health benefits of physical activity, there was a tendency to overall agreement with the evidence of benefits, although there was a proportion who agreed with deliberately fictitious statements that were included. This included 12% of participants who believed that activity could reduce the risk of glaucoma, 6% who believed that the only benefit of activity was weight loss, and 4% that thought physical activity could treat haemophilia. These statements were included to give an indication of positive affirmation bias to put the responses to other questions into context. Firm conclusions are difficult to draw from this, but it is possible that further professional education about the number and magnitude of health benefits of physical activity may be helpful, and the RCGP could take a lead on this given its announcement of physical activity being a clinical priority area.

There are a number of valid questionnaires which can be used to measure and monitor physical activity, however, this study showed limited awareness of these. Indeed, the two fictitious questionnaires included by the authors had similar levels of recognition as the real questionnaires that are already used in a variety of settings. Even simple single item questions, such as, ‘how many days in the last week have you achieved at least 30 minutes of moderate physical activity?’ are a place to start to raise the topic with patients. More robust measures are available, but simple measures may be sufficient to start with, especially given that lack of time is perceived as the main barrier to raising the issue of physical activity across all clinical groups.

The questions used in this study follow different formats, so the results from different sections are split into different tables to improve clarity. While at first glance this appears data-heavy, overall results are given, rather than scoring or weighting given to the questions, to improve clarity and reduce the risk of introducing further bias.

### Strengths and limitations

One issue with survey-type research such as this is that the response rate may influence results with responder bias. The response rate at each of the training sessions was not recorded, with the exception of the first training event, the other meetings were relatively small and the response rate was thought to be a high proportion of those in attendance. Response rate is an issue that needs to be considered in this context, as it may be that those with the least confidence or knowledge, who stand to gain the most from targeted education, may be least likely to complete the survey. In addition, these are questions asked at specific education events and it is not clear if these represent the views of the wider primary care team. A challenge is that validated questionnaires seeking understanding in this topic do not yet exist. Pragmatic decisions were made about questions used in this study, which have been developed from previous research in this area.

### Comparison with existing literature

While there have been numerous drives to raise awareness of the importance of physical activity, and options to promote activity, either by or through general practice, there has not been much recent work on primary healthcare team knowledge and confidence in this area. Previous research has focused on GP knowledge, but with the change in delivery with practice nurses now picking up much more of the routine chronic disease management, they may be best placed to advise at-risk patients. In addition, there has been little published work on the knowledge of GP registrars in this domain. By including these two key groups, this study covers a wider view of healthcare teams, and allows potential comparison between groups and identification of possible learning needs.

### Implications for practice

This study has shown that primary healthcare professionals do consider it a part of their role to discuss physical activity, and in many areas, there was a reasonable level of knowledge. However, barriers remain, and activity is far less commonly discussed with patients than other factors, such as smoking or blood pressure. The announcements by the RCGP to make physical activity a priority area will hopefully raise this topic in medical consciousness and stimulate further utilisation of this often-neglected area of primary and secondary prevention. The question that remains is whether continuing professional development in this health promotion area improves knowledge and promotion of physical activity within the primary care consultation, and whether this in turn leads to health benefits for the population served.
